# Recurrent Gestational Thrombocytopenia Affecting Four Consecutive Pregnancies: A Case Report

**DOI:** 10.7759/cureus.34005

**Published:** 2023-01-20

**Authors:** Ayooluwa K Omoloye, Kyi Pyar Than Yu, Satyanarayana V Sagi, Manjula Samyraju, Samson O Oyibo

**Affiliations:** 1 Internal Medicine, Peterborough City Hospital, Peterborough, GBR; 2 Diabetes and Endocrinology, Peterborough City Hospital, Peterborough, GBR; 3 Obstetrics and Gynecology, Peterborough City Hospital, Peterborough, GBR

**Keywords:** consecutive pregnancies, immune thrombocytopenia purpura, recurrent thrombocytopenia, normovolemic hemodilution, gestational thrombocytopenia

## Abstract

Gestational thrombocytopenia is the commonest cause of thrombocytopenia in pregnancy, accounting for 70-80% of cases. It is a benign condition that recovers completely in the postpartum period. Although the cause is not fully understood, it is thought that pregnancy-related hemodilution and increased platelet consumption play a significant contributory role. Several life-threatening causes of thrombocytopenia in pregnancy make up the remaining 20-30% of cases. There are reports of recurrence of GT in women, but not many case reports document more than three consecutive episodes. We present a case of a young woman who had gestational thrombocytopenia during the third trimester of her four consecutive pregnancies, followed by complete recovery after each pregnancy. We ruled out other sinister and life-threatening causes of thrombocytopenia in pregnancy. During her four consecutive pregnancies, her platelet count fell below normal at the beginning of the third trimester, with a nadir towards the end. The other hematological indices, generally used as markers of hemodilution, remained in the normal pregnancy-specific reference range during each of her four pregnancies. This case argues against pregnancy-related hemodilution being a major factor in gestational thrombocytopenia. A systematic approach is paramount when differentiating the several causes of thrombocytopenia in pregnancy.

## Introduction

Thrombocytopenia is a blood platelet count below 150 x 10^9^/L. It is classified according to the platelet count as mild (100-150 x 10^9^/L), moderate (50-100 x 10^9^/L), or severe (< 50 x 10^9^/L). Thrombocytopenia complicates 7-10% of all pregnancies [1]. Gestational thrombocytopenia (GT) is the commonest cause of thrombocytopenia in pregnancy, accounting for 70-80% of cases. GT is a benign condition that recovers completely postpartum [1]. The cause of GT is not fully understood, but hemodilution due to increased plasma volume, increased peripheral platelet consumption, hypersplenism, platelet aggregation, reduced production, and pregnancy-induced immune thrombocytopenia have all been suggested [1].

Other more life-threatening causes of thrombocytopenia in pregnancy make up the remaining 20-30%. These include pre-eclampsia/eclampsia, hemolytic-elevated-liver-enzymes-low-platelet (HELLP) syndrome, acute fatty liver of pregnancy, immune thrombocytopenic purpura (ITP), thrombotic thrombocytopenic purpura, hemolytic uremic syndrome, autoimmune diseases (lupus, antiphospholipid syndrome), infections (hepatitis, human immunodeficiency virus, sepsis), disseminated intravascular coagulation, drugs (heparin), vitamin B12/folate deficiency, bone marrow dysfunction and hypersplenism [1].

GT is a diagnosis of exclusion. However, a systematic approach is paramount when differentiating the several causes of thrombocytopenia in pregnancy. This will help prevent over-investigating and causing undue anxiety for women with GT. Patients with GT characteristically have mild thrombocytopenia (100-150 x 10^9^/L) in the third trimester that recurs in subsequent pregnancies, spontaneous recovery soon after delivery, normal platelet counts when not pregnant, and no associated conditions that could otherwise account for the thrombocytopenia [2,3]. Additionally, the fetal platelet count is usually normal.

There are reports of recurrence of GT in women, but not many case reports document more than three consecutive episodes. We present a case of a young woman who had GT during the third trimester of four consecutive pregnancies, with complete recovery after each pregnancy.

## Case presentation

Medical history and demographics

A 29-year-old woman had a coincidental finding of mild thrombocytopenia during a routine joint obstetric/endocrinology clinic visit. She was 28 weeks into her fourth pregnancy (gravida 4, para 3). She had no symptoms on systemic review. She had no history of menorrhagia, easy bruising, or bleeding such as epistaxis or gingival bleeding. Her medical history included Graves’ hyperthyroidism and sickle cell trait. She had a history of low platelet count in her three previous pregnancies. There was no history of low platelet count before her first pregnancy. Her medication list included omeprazole, ferrous sulfate, and prenatal vitamins. She was not taking any medication that could cause thrombocytopenia and had no drug allergies. She was an ex-smoker, rarely drank alcohol, and she had no family history of bleeding or bruising disorders. On examination, she had no signs of bleeding or bruising besides signs of a gravid uterus. She had normal blood pressure. There was no ankle edema (she had never had ankle edema in her previous pregnancies).

Investigations

The hematological investigation demonstrated a low platelet count of 116 x 10^9^/L at 28 weeks gestation (her platelet counts were in the normal range during the first and second trimesters of this pregnancy). Her red blood cell count, white blood cell count, hemoglobin, and hematocrit (packed cell volume) values were all within the pregnancy-specific reference range (Table 1).

**Table 1:** Hematological results at 28 weeks gestation demonstrating mild thrombocytopenia

Hematological indices	Results	Pregnancy-specific reference range
Hemoglobin (g/L)	127	100-150
Hematocrit (L/L)	0.379	0.32-0.46
Red blood cell count (10^12^/L)	4.61	3.5-5.0
Platelet count (10^9^/L)	116	150-400
White cell count (10^9^/L)	8.7	4.0-15.0

Her reticulocyte count (percentage) and coagulation profile (fibrinogen, prothrombin time, and activated partial thromboplastin clotting time) were normal. A hemoglobinopathy screen revealed sickle cell trait and no evidence of thalassemia. Blood grouping indicated group B-negative. Blood film examination did not reveal abnormalities such as red blood cell fragmentation, platelet aggregation, or clumping. The biochemical investigation demonstrated normal renal, liver, and thyroid function. Serum albumin, calcium, and vitamin D levels were normal. Serum ferritin was slightly low at 29 µg/L (normal range: 30-400), and her serum vitamin B12 and folate levels were normal. Serum electrophoresis was normal. Immunological investigation revealed that the serum immunoglobulins were within the third-trimester reference range. Anti-cardiolipin, anti-nuclear, anti-beta-2-glycoprotein-1, and antiphospholipid antibodies were all negative. Her anti-thyroid peroxidase antibody levels were slightly raised at 92 IU/ml (normal range: < 34). Her thyroid receptor antibodies were negative, albeit positive, at the initial diagnosis of hyperthyroidism. A screen for the hepatitis virus, human immunodeficiency virus, and syphilis were negative. A previous abdominal ultrasound examination demonstrated a normal-sized liver and spleen.

Historical results demonstrate thrombocytopenia occurring during her previous three pregnancies. The onset was always at 28 weeks, with a nadir occurring between 37 to 40 weeks. Liver enzymes were normal during the three previous pregnancies. A coagulation profile was available for the third pregnancy, which was normal. She had normal platelet counts after each pregnancy, albeit the platelet counts were checked late after the first and second pregnancies (Table 2).

**Table 2:** The onset and course of gestational thrombocytopenia during the patient’s three previous pregnancies The onset and course were similar in all three previous pregnancies.

Pregnancy	Onset of thrombocytopenia		Onset of nadir		Documented normal platelet count
	Platelet count (x 10^9^/L)	Gestation (weeks)	Platelet count (x 10^9^/L)	Gestation (weeks)	Post-delivery (weeks)
1^st^	108	28	106	40	52
2^nd^	128	28	91	39	72
3^rd^	139	28	113	37	3

Treatment

A diagnosis of gestational thrombocytopenia was explained to the patient. The patient did not have any specific treatment apart from regular platelet count monitoring during the pregnancy. She was told that gestational thrombocytopenia is generally harmless and likely to recur during the third trimester of future pregnancies.

Outcome and follow-up

Platelet counts were monitored throughout the rest of her pregnancy. Her platelet count dropped to a nadir of 100 x 10^9^/L at 38 weeks gestation (hemoglobin and liver enzymes normal), then climbed up to 111 x 10^9^/L at 39 weeks gestation. She had an uneventful delivery of a baby girl at 40 weeks gestation. A blood count performed because of mild postpartum hemorrhage (700 ml) demonstrated a normal hemoglobin level and a platelet count of 121 x 10^9^/L. A follow-up test revealed a platelet count in the normal range (197 x 10^9^/L) during the second week (nine days) post-delivery.

**Figure 1:**
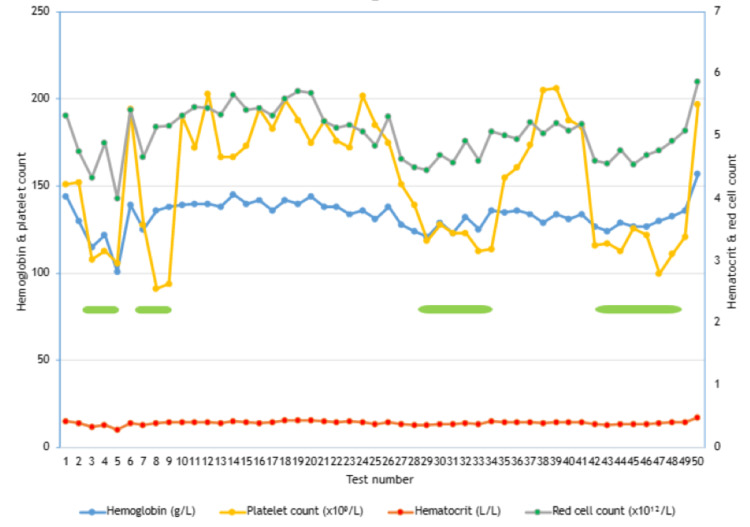
Graphical representation of patient’s hematological indices during her four consecutive pregnancies over 12 years. Platelet counts remained below the pregnancy-specific reference range during the third trimester of each of her four pregnancies, while the other hematological indices (markers of hemodilution) remained within the normal range. The green bars indicate the third trimester period of each of the four consecutive pregnancies.

Even though the patient’s platelet counts were consistently low during the third trimester of each of her four consecutive pregnancies, the other hematological indices always remained within the pregnancy-specific reference range (Figure 1).

## Discussion

Although gestational thrombocytopenia (GT) is generally a benign condition characterized by complete platelet recovery in the postpartum period, affected patients are at risk of excessive investigation or over-treatment for, or a delay in diagnosis or misdiagnosis of a more sinister cause of thrombocytopenia in pregnancy. Affected patients are also at risk of being denied regional anesthesia during labor, given the current lack of knowledge regarding platelet function and expected kinetics in GT [4]. Therefore, GT can be a diagnostic and management dilemma until complete recovery has been confirmed in the postpartum period.

The platelet count is usually above 100 x 10^9^/L in patients with GT, but cases of moderate/severe thrombocytopenia have been reported. One such case report described a woman with severe thrombocytopenia who was treated as a case of ITP with blood transfusion and steroid therapy. Only when there was complete recovery soon after delivery was the diagnosis changed to GT [5]. There are no reliable laboratory methods to differentiate between gestational thrombocytopenia and ITP during pregnancy. Both conditions can have raised platelet antibodies, and both diagnoses are made by exclusion of other causes [6]. ITP should be suspected based on a history of bleeding, low platelet count prior to pregnancy, a platelet count below 70 x 10^9^/L in the first or second trimester of pregnancy, and the presence of immature platelets on blood film examination [7]. The woman in our case report had GT in four consecutive pregnancies, with an episode of moderate thrombocytopenia occurring in her second pregnancy. She did not require any treatment and did not have any delivery complications.

A meta-analysis of longitudinal studies demonstrated that plasma volume increased by 3-9% during the first trimester of pregnancy, by 12-36% during the second trimester, and by 38-51% during the third trimester of pregnancy. This hemodilution contributes to a drop in hemoglobin levels, red blood cell count, and hematocrit (packed cell volume) levels throughout pregnancy. Other nutritional biomarkers are affected; however, hemoglobin levels, red cell count, and hematocrit levels are the main markers of hemodilution in pregnancy [8,9]. Our patient had similar drops in platelet count during her four consecutive pregnancies. There were slight but insignificant drops in the hematological markers of hemodilution (e.g., hemoglobin, hematocrit, red cell count), but levels remained in the normal pregnancy-specific range throughout her four pregnancies. This suggests that hemodilution did not significantly contribute to GT in this case.

The results of a large case-control study of over 3,500 pregnancies have challenged the hemodilution theory [4]. Plasma volume expansion and resultant hemodilution affect all pregnancies, but GT was present in only 12% of pregnancies. This condition appears to be patient-specific rather than pregnancy-specific. Additionally, the slight drop in the hemoglobin level did not match the large drop in the platelet count [4]. This same study also found a greater increase in the mean platelet size in patients with GT compared to controls, suggesting the presence of increased platelet turnover in patients with GT. This study also found that the platelet counts rapidly improved within a week after delivery, making an autoimmune mechanism very unlikely. The authors concluded that GT could not be explained by hemodilution from an expanded plasma volume, and an autoimmune process is unlikely. Instead, GT is likely due to increased platelet turnover associated with the high-shear, uteroplacental blood flow during the third trimester in genetically susceptible women [4]. More research is required to explore platelet kinetics and genetic susceptibility in patients with GT.

Our case report demonstrates that hemodilution is not a major contributor to GT. Although each thrombocytopenic episode was mild, occurring at the beginning of the third trimester, and the nadir occurring at the end of each pregnancy with documented recovery after each pregnancy, the magnitude of the drop in platelet count was always in excess compared to any corresponding drop in the usual hematological markers of hemodilution. The markers of hemodilution (hematocrit, hemoglobin, red cell count) remained within the pregnancy-specific reference range throughout the four consecutive pregnancies.

There are rare reports of thrombocytopenia in patients using proton pump inhibitors, which have been specific to pantoprazole [10]. Our patient was taking omeprazole just before and during her fourth pregnancy, and there was no history of using any proton pump inhibitors during her previous pregnancies. While taking omeprazole, her platelet count followed the same pattern as in her three previous pregnancies, ruling out omeprazole as a contributory factor.

There are several reports of recurrence of GT in women; however, we did not find any case report documenting more than three consecutive episodes. A previous case report described severe GT occurring in two consecutive pregnancies, with both episodes discovered late (after 35 weeks gestation) [11]. There was a complete recovery of the platelet count soon after delivery. Despite a previous history of GT, the woman still required extensive investigation and strict feto-maternal surveillance, just in case the thrombocytopenia was secondary to another life-threatening cause [11]. Another case report described moderate thrombocytopenia occurring in two consecutive pregnancies, discovered after 35 weeks gestation. The patient had a one-unit platelet transfusion in the first pregnancy, and both her pregnancies had no bleeding complications [12]. This case reports and similar reports of recurrence suggest that GT can present a diagnostic and management dilemma even in women with a documented history of GT.

Though the consensus suggests that no specific therapy is required for GT, there is still variable concern among obstetricians and anesthetists around regional anesthesia and delivery when faced with lower platelet counts or moderate/severe thrombocytopenia [13]. During the antenatal period, it is advisable to monitor platelet count every four weeks in women with a history of gestational thrombocytopenia. More frequent monitoring may be required during the third trimester [13]. The importance of awareness and national and local recommendations should be considered.

## Conclusions

We have presented a case of recurrent GT, with each episode lasting throughout the third trimester of four consecutive pregnancies. The platelet counts were below the pregnancy-specific reference range, while the hematological markers of hemodilution remained within the reference range. This case supports the theory that GT is more than just a case of pregnancy-related hemodilution. 

Women with GT can present with lower platelet counts, prompting suspicion of other life-threatening causes of thrombocytopenia in pregnancy. As a result, some patients may still have to undergo extensive investigation and antenatal monitoring. Using a systematic approach to differentiate the several causes of thrombocytopenia in pregnancy will help prevent over-investigating and causing undue anxiety for women with GT.
